# Increased Nucleus Accumbens Connectivity in Resting-State Patients With Drug-Naive, First-Episode Somatization Disorder

**DOI:** 10.3389/fpsyt.2019.00585

**Published:** 2019-08-16

**Authors:** Yangpan Ou, Qinji Su, Feng Liu, Yudan Ding, Jindong Chen, Zhikun Zhang, Jingping Zhao, Wenbin Guo

**Affiliations:** ^1^Department of Psychiatry, The Second Xiangya Hospital of Central South University, Changsha, China; ^2^National Clinical Research Center on Mental Disorders, Changsha, China; ^3^Mental Health Center, The First Affiliated Hospital of Guangxi Medical University, Nanning, China; ^4^Department of Radiology, Tianjin Medical University General Hospital, Tianjin, China

**Keywords:** somatization disorder, functional connectivity, reward circuit, functional magnetic resonance imaging, support vector machine

## Abstract

The nucleus accumbens (NAc) plays an important role in the reward circuit, and abnormal regional activities of the reward circuit have been reported in various psychiatric disorders including somatization disorder (SD). However, few researches are designed to analyze the NAc connectivity in SD. This study was designed to explore the NAc connectivity in first-episode, drug-naive patients with SD using the bilateral NAc as seeds. Twenty-five first-episode, drug-naive patients with SD and 28 healthy controls were recruited. Functional connectivity (FC) was designed to analyze the images. LIBSVM (a library for support vector machines) was used to identify whether abnormal FC could be utilized to discriminate the patients from the controls. The patients showed significantly increased FC between the left NAc and the right gyrus rectus and left medial prefrontal cortex/anterior cingulate cortex (MPFC/ACC), and between the right NAc and the left gyrus rectus and left MPFC/ACC compared with the controls. The patients could be separated from the controls through increased FC between the left NAc and the right gyrus rectus with a sensitivity of 88.00% and a specificity of 82.14%. The findings reveal that patients with SD have increased NAc connectivity with the frontal regions of the reward circuit. Increased left NAc-right gyrus rectus connectivity can be used as a potential marker to discriminate patients with SD from healthy controls. The study thus highlights the importance of the reward circuit in the neuropathology of SD.

## Introduction

Somatization disorder (SD) is a psychiatric disorder characterized by multiple, recurrent, and clinically significant complaints of somatic symptoms. Patients with SD usually undergo numerous medical examinations without an accurate diagnosis. Consequently, their medical cost increases dramatically (1).

In recent years, neuroimaging techniques provide us with new ways to analyze changes of brain function and structure in psychiatric patients (2). Anatomical alterations and connectivities have been revealed in patients with SD using structural imaging techniques. For example, patients with SD showed decreased fractional anisotropy in the right cingulum and right inferior fronto-occipital fasciculus (3). Atmaca et al. found that patients with SD had significantly small amygdala relative to controls (4). By contrast, increased bilateral caudate nuclei volumes have been detected in patients with SD compared with controls (5).

Previously, abnormal brain regional activities have been found in SD using functional neuroimaging methods. For example, patients with SD showed increased coherence-based regional homogeneity (Cohe-ReHo) in the left medial prefrontal cortex/anterior cingulate cortex (MPFC/ACC) (6), and increased regional activity in the bilateral MPFC has been detected in patients with SD (7). Patients with SD also showed abnormal functional connectivity (FC) between the cingulate-insular network and sensorimotor network (SMN)/anterior default-mode network (DMN), between the posterior DMN and SMN, and between the anterior DMN and posterior DMN/SMN compared with healthy controls (8). Increased FC strength in the right inferior temporal gyrus (ITG) has been found in patients with SD (9). Moreover, patients with SD exhibited increased cerebellar-DMN connectivity, which was correlated to the somatization severity and personality (10). However, little attention has been focused on the dysconnectivity of the reward circuit in SD.

The reward circuit is a group of neural structures related to associative learning, incentive salience, and positive emotions (11). The mesolimbic reward circuit comprises the NAc, ventral tegmental area (VTA), prefrontal cortex (PFC), and hippocampus (12, 13). Located in the ventral striatum, the NAc is an important brain reward region that integrates different inhibitory and excitatory inputs to salience signal of rewarding stimuli (14). In a previous study, patients with SD presented hypoperfusion in the frontal and prefrontal areas using the single-photon emission computed tomography (SPECT) scan (15). Moreover, Hakala et al. revealed regional cerebral hypometabolism in the caudate nuclei, right precentral gyrus, and left putamen in patients with SD (16). These findings suggest that reward circuit is involved in the pathophysiology of SD.

SVM (support vector machine) is a supervised learning model with correlated learning algorithms that analyzes data used for regression and classification analysis (17). Given a pieces of training examples, an SVM training algorithm creates a model that deals new examples to one sort or the other, making it a non-probabilistic binary linear classifier. SVM structures a hyperplane or set of hyperplanes in a high- or infinite-dimensional space, which can be applied for regression, classification, or other roles like outlier detection. In particular, SVM utilizes a training dataset to get differences between the patients and the controls, and a testing dataset is used to assess classification performance on uncharted data. The classifier algorithm is applied with a leave-pair-out cross-validation (LPO-CV) method to acquire the highest specificity and sensitivity (18). SVM has been widely performed in medical disease. For example, SVM was applied to identify patients with coronary heart disease (CHD) from non-CHD individuals (19). Wang et al. revealed that SVM model could diagnose lymph node metastasis better than preoperative short axis size of largest lymph node on computed tomography (20). In our previous study, SVM analysis could be used to discriminate patients with SD from healthy controls with proper sensitivity and specificity (6). In this study, SVM was used to examine whether abnormal NAc connectivity could be applied to distinguish the patients from the controls.

So far, few studies have analyzed abnormal FC of the reward circuit in SD using the seed-based FC method, which is conducted by calculating the correlations between the preselected brain regions (seeds) and the rest brain regions. This method has been used in subjects with high social anhedonia, and the cortico-striatal abnormalities in the reward-related symptomatology have been revealed (21). In this study, we employed bilateral NAc (from the Harvard Oxford Atlases) as seeds. Then, the seed-based FC method was used to identify abnormal connectivity between the seeds and other regions of brain. Based on abovementioned findings, we hypothesized that increased NAc connectivity would be detected in SD, particularly within the reward circuit, which could be used to discriminate the patients from the controls. We also expected there were some correlations between abnormal FCs and clinical variables in the patients.

## Materials and Methods

### Participants

Twenty-five right-handed patients with first-episode and drug-naive SD were recruited from the First Affiliated Hospital of Guangxi Medical University. Twenty-eight healthy controls were recruited from the community. The controls were screened by using the Structured Clinical Interview of the Diagnostic and Statistical Manual of Mental Disorders-IV (SCID), non-patient edition (22), and no neuropsychiatric disorders in their first-degree relatives. Patients with SD should meet the criteria of the SCID, patient edition (22). Somatic symptoms of patients with SD should originate from several speciﬁc origins (i.e., at least four pain symptoms, two gastro-intestinal symptoms, one sexual symptom, and one pseudo-neurological symptom), and the symptoms were in the absence of a medical explanation, factitious disorder, or malingering (23). Participants were excluded according to the following criteria: other psychiatric disorders (e.g., bipolar disorders, schizophrenia, or personality disorders), severe medical diseases, substance abuse disorders, mental retardation, and any limits for MRI.

The Hamilton Anxiety Scale (HAMA) (24), Hamilton Depression Scale (HAMD, 17 items) (25), and somatization subscale of Symptom Checklist-90 (SCL-90) (26) were used to assess the symptomatic severity of anxiety, depression, and somatization. Eysenck Personality Questionnaire (EPQ) (27) was used to evaluate personality dimensions. Wisconsin Card Sorting Test (WCST) (28) and digit symbol coding of Wechsler Adult Intelligence Scale (WAIS) were applied to identify cognitive functions.

After given detailed knowledge of the contents, all the participants signed a written informed consent. The local ethics committee of the First Affiliated Hospital of Guangxi Medical University approved this study.

### MRI Acquisition

Functional MRI scans were obtained with a Siemens 3T scanner. During the procedures, the participants were asked to remain motionless and awake with their eye closed. Soft earplugs and foam pads were used to reduce scanner noise and head motion. Resting-state functional scans were obtained with a gradient-echo echo-planar imaging sequence using the following parameters: repetition time/echo time = 2,000/30 ms, 30 slices, 64 × 64 matrix, 90° flip angle, 24-cm FOV, 4-mm slice thickness, 0.4-mm gap, and 250 volumes (500 s).

### Data Preprocessing

We preprocessed the imaging data with Data Processing & Analysis for (resting-state) Brain Imaging (29) in MATLAB. Slice timing and head movement were first corrected, and no participant had more than 2 mm of maximal displacement in any direction of x, y, and z and more than 2° in any angular dimension. After that, the images were normalized in the standard Montreal Neurological Institute (MNI) EPI space and resampled with 3×3×3-mm^3^ resolution. The obtained images were then smoothed with a 4-mm full width at half-maximum Gaussian kernel, bandpass filtered (0.01–0.08 Hz), and linearly detrended. In addition, framewise displacement (FD) was computed as described in a previous study (30). The mean FD is a covariate of no interest to handle the residual effects caused by head motion. We removed time points with FD > 0.2mm to control aggressive head motion. We did not regress out the global signal since it was suggested to be saved in processing the FC data (31).

### FC Processing

Bilateral NAc from the Harvard Oxford Atlases were selected as seeds for the whole-brain FC processing with the software REST (32). For each participant, seed-based FC was computed as Pearson correlation coefficients between the seeds and other voxels of the whole brain. The correlation coefficients were then z-transformed for standard purpose, and seed-based FC maps were generated.

### Statistical Analysis

Two-sample *t* tests were performed to compare the distribution of age, years of education, and clinical scales between patients with SD and healthy controls. A chi-square test was used to judge sex distributions.

Group differences were compared using voxel-wise two sample t-tests. Age and the mean FD values were used as covariates to minimize the potential effects of these variables. The significance level was set at *p* < 0.05 for multiple comparisons corrected by Gaussian random field (GRF) theory (voxel significance: *p* < 0.001, cluster significance: *p* < 0.05).

LIBSVM (33) was performed to examine whether abnormal FC between bilateral NAc and other brain regions could distinguish patients with SD from healthy controls.

To explore the correlations between abnormal FC values and clinical variables, voxel-based correlations were conducted. The correlation results were Bonferroni corrected at *p* < 0.05.

## Results

### Characteristics of the Participants

General information of the participants is shown in **Table 1**, and no difference was observed regarding age, sex ratio, education level, EPQ extraversion/lie scores, digit symbol coding of WAIS, and WCST between the two groups. The scores of HAMA, HAMD, EPQ psychoticism/neuroticism, and somatization subscale of SCL-90 of the patients were higher than those of the controls (**Table 1**).

**Table 1:** Characteristics of participants.

Variables	Patients (n = 25)	Controls (n = 28)	*p* value
Age (years)	41.00 ± 10.76	38.71 ± 9.59	0.42^b^
Sex (male/female)	4/21	6/22	0.73^a^
Years of education (years)	7.72 ± 4.39	7.82 ± 2.59	0.92^b^
FD (mm)	0.08 ± 0.03	0.10 ± 0.05	0.02^b^
Illness duration (months)	59.12 ± 62.22		
Somatization subscale of SCL-90	28.48 ± 10.37	14.32 ± 3.44	<0.001^b^
HAMD	18.84 ± 7.31	2.60 ± 1.83	<0.001^b^
HAMA	22.96 ± 10.95	0.53 ± 0.99	<0.001^b^
Digit symbol coding of WAIS	8.28 ± 2.87	9.64 ± 2.15	0.06^b^
EPQ			
Extraversion	46.84 ± 11.02	49.75 ± 9.65	0.31^b^
Psychoticism	50.52 ± 9.01	45.00 ± 8.54	0.03^b^
Neuroticism	57.36 ± 9.18	46.78 ± 10.24	<0.001^b^
Lie	49.44 ± 12.31	47.96 ± 11.01	0.65^b^
WCST			
Number of categories achieved	3.52 ± 1.76	3.89 ± 1.66	0.43^b^
Number of errors	22.84 ± 9.12	24.71 ± 8.91	0.45^b^
Number of perseverative errors	20.04 ± 9.48	22.82 ± 8.72	0.27^b^

a The p value for sex distribution was obtained by a chi-square test.

b The p values were obtained by two samples t-tests.

FD, Framewise displacement; HAMD, Hamilton depression scale; HAMA, Hamilton Anxiety Scale; SCL-90, Symptom Checklist-90; EPQ, Eysenck Personality Questionnaire; WAIS, Wechsler Adult Intelligence Scale; WCST, Wisconsin Card Sorting Test.

### Group Differences in Seed-Based FC Analyses

**Figure 1:**
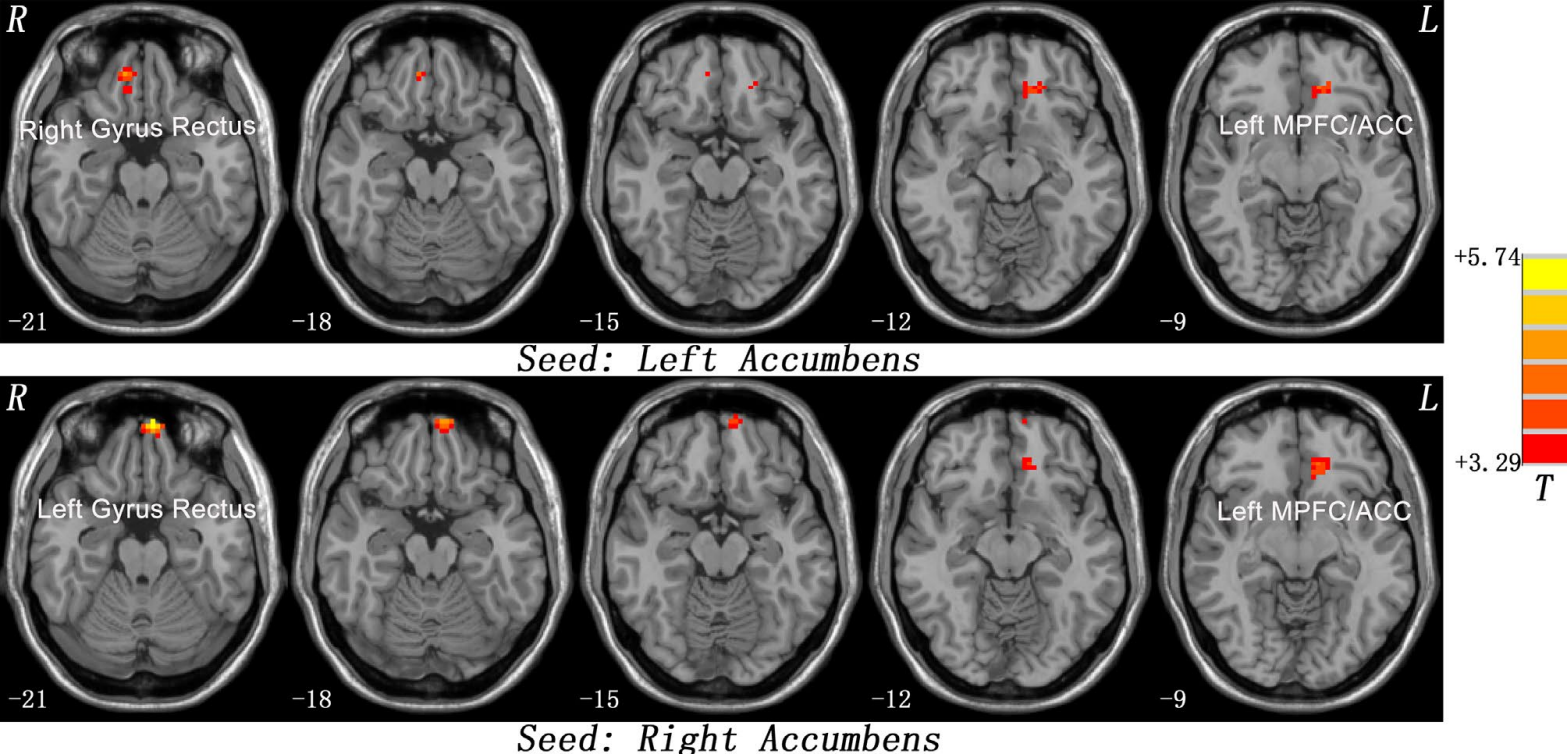
Statistical maps showing seed-based functional connectivity differences between subject groups. The patients showed significantly increased FC between the left NAc and the right gyrus rectus and left MPFC/ACC, and between the right NAc and the left gyrus rectus and left MPFC/ACC compared with the controls. Red denotes high FC values in the patients, and the color bar indicates the T values from two-sample t-tests. FC, functional connectivity; NAc, nucleus accumbens; MPFC/ACC, medial prefrontal cortex/anterior cingulate cortex.

The patients showed significantly increased FC between the left NAc and the right gyrus rectus (*t* = 4.2239, *p* < 0.001) and left MPFC/ACC (*t* = 3.9208, *p* < 0.001), and between the right NAc and the left gyrus rectus (*t* = 5.7374, *p* < 0.001) and left MPFC/ACC (*t* = 4.3168, *p* < 0.001) compared with the controls (**Figure 1** and **Table 2**).

**Table 2:** Regions with increased functional connectivity with the accumbens in patients.

Cluster location	Peak (MNI)			Number of voxels	*T* value
	x	y	z		
*Seed: Left Accumbens*					
Right Gyrus Rectus	12	45	−24	38	4.2239
Left MPFC/ACC	−12	36	−9	25	3.9208
*Seed: Right Accumbens*					
Left Gyrus Rectus	−6	63	−21	38	5.7374
Left MPFC/ACC	−12	36	−9	39	4.3168

MNI, Montreal Neurological Institute; MPFC, medial prefrontal cortex; ACC, anterior cingulate cortex.

### Correlations Between Abnormal FC and Clinical or Personality or Cognitive Variables in the Patients

No correlations were detected between increased FC between the left NAc and the right gyrus rectus and left MPFC/ACC, and between the right NAc and the left gyrus rectus and left MPFC/ACC and clinical or personality or cognitive variables (WCST and digit symbol coding of WAIS) in the patients.

### LIBSVM Analysis

**Figure 2:**
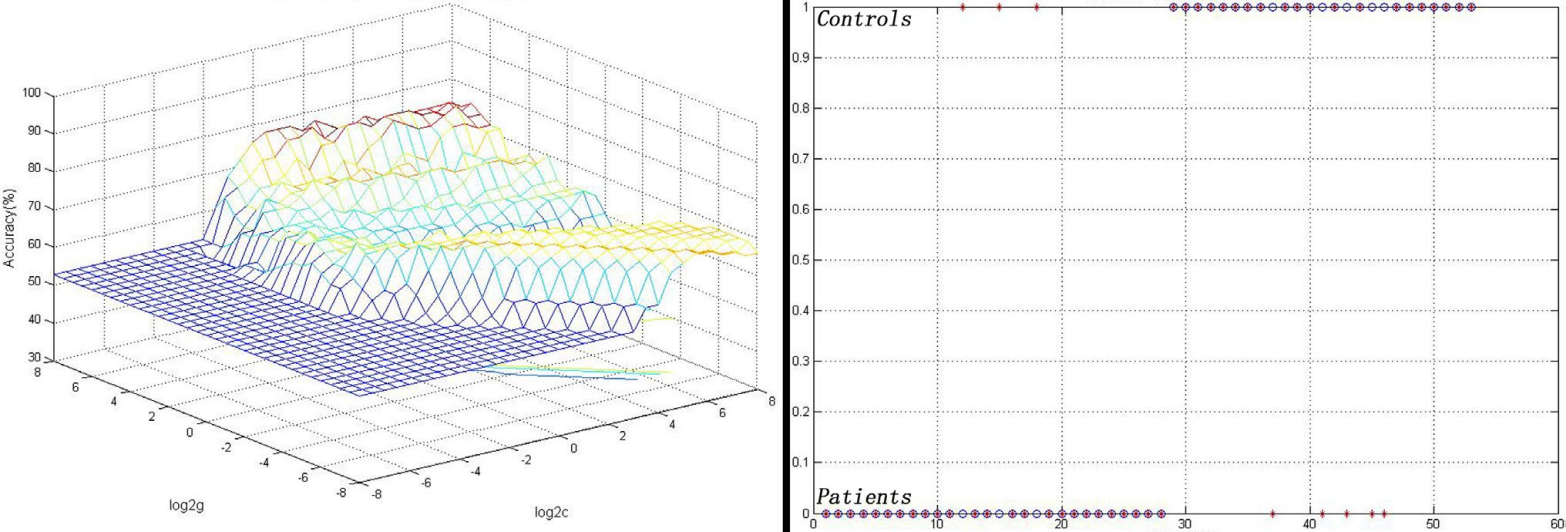
Visualization of the SVM results for identifying patients from controls using the FC values between the left NAc and the right gyrus rectus. Left: 3D view of the classified accuracy with the best parameters; right: classified map of the FC values between the left NAc and the right gyrus rectus. SVM, Support vector machine; FC, functional connectivity; NAc, nucleus accumbens.

As shown in **Figure 2**, the FC values between the left NAc and the right gyrus rectus could correctly classify 22 of 25 patients and 23 of the 28 controls, resulting in an optimal sensitivity of 88.00% and an optimal specificity of 82.14% (**Figure 2**).

## Discussion

In this study, we used bilateral NAc as seeds to analyze the seed-based FC in first-episode and drug-naive SD. The primary finding is that patients showed significantly increased FC values between the left NAc and the right gyrus rectus and left MPFC/ACC, and between the right NAc and the left gyrus rectus and left MPFC/ACC compared with the controls. Increased connectivity between the left NAc and the right gyrus rectus can be used as a potential marker to discriminate patients with SD from healthy controls with optimal sensitivity and specificity. There are no correlations between abnormal FC values and clinical variables in the patients.

Increased NAc connectivity with other brains have been found in this study. The NAc receives heterogeneous gamma-aminobutyric acid (GABAergic) and dopaminergic projections from the VTA (34, 35) as well as glutamatergic afferents from the PFC (36), hippocampus (37, 38), thalamus (39), and amygdala (40). The NAc is a complex, integral hub in the reward circuit (41). For example, patients with SD commonly have pain symptoms, and the NAc plays an important role in reward-aversion processing during pain perception (42). Baliki et al. found that the NAc showed abnormal activities when patients were in the presence of chronic pain, and the NAc activity could anticipate analgesic potential on chronic pain (43).

The MPFC/ACC plays an important role in the reward circuit, which generates emotional and cognitive information (44), and abnormal activity within the MPFC areas may be related to augment pain perception in patients with SD (45). Furthermore, a study showed that negative emotional stimuli could activate the MPFC/ACC, which revealed that the MPFC/ACC might be involved in appraisal and expression of negative emotion (46).

The gyrus rectus, also named straight gyrus, is located at the medial most margin of the inferior surface of frontal lobe and is continuous with the superior frontal gyrus on the medial surface. Up to now, the function of the gyrus rectus is unclear. However, a research suggested that patients with obsessive-compulsive disorder have decreased prefrontal hemodynamic response (47). In our study, patients with SD showed significantly increased FC values between the left NAc and the right gyrus rectus and left MPFC/ACC, and between the right NAc and the left gyrus rectus and left MPFC/ACC compared with the controls.

Increased FC is usually considered as compensatory reallocation or dedifferentiation to functional deficits in the brain regions (48, 49). Patients with SD may have deficits in emotional processing, and the MPFC/ACC is related to the negative emotion (46). Du et al. found that the stimulated dorsolateral PFC-NAc FC can predict the anti-depressant and anti-anxiety effects of repeated transcranial magnetic stimulation (rTMS) (50). Furthermore, deep brain stimulation (DBS) targeting the NAc and rTMS about the left dorsolateral PFC also exhibited antidepressant and antianxiety effects (51–53). Therefore, increased NAc connectivity in the present study may be a compensatory effort to functional deﬁcits in these regions.

In a previous study, a significantly positive correlation has been found between increased activity in the bilateral superior MPFC and the somatization subscale scores of SCL-90 in patients with SD (7). We hypothesized that correlations would be detected between increased NAc connectivity and clinical parameters. Therefore, no correlation in the present study is somewhat surprised. There are several possibilities account for this issue. First, sample size of this research may be small to establish a correlation. Second, increased NAc connectivity may be an internal alteration for patients with SD independent of symptomatic severity. Third, the clinical parameters are concentrated, such as the scores of the digit symbol coding of WAIS of the patients with SD are centered at 8.28 points.

SVM analysis suggests that the increased FC values between the left NAc and the right gyrus rectus could be used to discriminate patients with SD from healthy controls with a sensitivity of 88.00% and a specificity of 82.14%. A highly credible research is characterized by specificity and sensitivity above 70% in the medical domain (54). Interpretation of the high discriminative power result must think about the multivariate nature of the SVM method. SVM, a multivariate method, has been additionally based on inter-regional correlations, while standard quality univariate techniques regard each voxel as a spatially independent unit (55). Therefore, increased FC values may be used as a potential marker to discriminate patients with SD from healthy controls.

Our study has several limitations. First, this research is a cross-sectional one, and it is unclear how the NAc connectivity will alter after treatment. A longitudinal study is needed to clarify this issue. Second, some studies showed that abnormal FC was correlated to anhedonia (56). However, psychological tests about anhedonia were not assessed in this study. The relationship between abnormal FC and anhedonia remains unknown. Third, the sample size in our study is relatively small, which may minimize the translational value of our findings. Fourth, the HAMA scores and HAMD scores were significantly different between the SD group and HC group. Therefore, there is a possibility that the present findings may be affected by the HAMA scores and HAMD scores. To clarify this issue, we reanalyzed the data with age, mean FD values, HAMA scores, and HAMD scores as covariates and obtained similar results as previously reported. Therefore, the present findings seemed impossible to be affected by HAMA scores and HAMD scores. Finally, the confounding effects of scans, such as respiratory and cardiac rhythm, could not be completely eliminated.

Despite the limitations, the current research first examines the NAc connectivity in resting-state patients with first-episode, drug-naive SD. The findings reveal that patients with SD have increased NAc connectivity with some regions of the reward circuit. Increased NAc connectivity can be used as a potential marker to discriminate patients with SD from healthy controls. This study thus highlights the importance of the reward circuit in the neuropathology of SD.

## Data Availability

All datasets generated for this study are included in the manuscript and/or the **Supplementary files**.

## Author Contributions

WG and JZ designed the study. WG, FL, QS, and ZZ collected the original imaging data. WG, FL, YD, and JC managed and analyzed the imaging data. YO wrote the first draft of the manuscript. All the authors contributed to and approved the final manuscript.

## Funding

This study was supported by grants from the National Key R&D Program of China (Grant Nos. 2016YFC1307100 and 2016YFC1306900) and the National Natural Science Foundation of China (Grant Nos. 81571310, 81771447, and 81630033).

## Conflict of Interest Statement

The authors declare that the research was conducted in the absence of any commercial or financial relationships that could be construed as a potential conflict of interest.
