# Supplementary material for: Increased Nucleus Accumbens Connectivity in Resting-State Patients With Drug-Naive, First-Episode Somatization Disorder
Source: Front Psychiatry. 2019 Aug 16;10:585. doi: 10.3389/fpsyt.2019.00585 (PMC6706814; doi:10.3389/fpsyt.2019.00585)
Supplement: Supplementary file 1 [file DataSheet_1.docx]

Supplementary Material

# Supplementary Data

Regions with increased functional connectivity with age, mean FD values, HAMA scores, and HAMD scores as covariates

| Cluster location | Peak (MNI) | | | Number of voxels | *T* value |
| --- | --- | --- | --- | --- | --- |
|  | x | y | z |  |  |
| *Seed: Left Accumbens* |  |  |  |  |  |
| Right Gyrus Rectus | 12 | 45 | -24 | 33 | 4.1830 |
| Left MPFC/ACC | -18 | 36 | -12 | 23 | 3.9245 |
|  |  |  |  |  |  |
| *Seed: Right Accumbens* |  |  |  |  |  |
| Left Gyrus Rectus | -6 | 63 | -21 | 34 | 5.6086 |
| Left MPFC/ACC | -12 | 36 | -9 | 38 | 4.2773 |

MNI = Montreal Neurological Institute; MPFC = medial prefrontal cortex; ACC = anterior cingulate cortex
